# A predictive model of the sunscreen use in the paddy workers based on the health action process approach model: a path analysis

**DOI:** 10.1186/s42506-020-00053-y

**Published:** 2020-09-21

**Authors:** Hadiseh Panahi, Leili Salehi, Zohreh Mahmoodi

**Affiliations:** 1 Department of Health Education & Promotion, School of Health, Alborz University of Medical Sciences, Karaj, Iran; 2 Research Center for Health, Safety and Environment, Alborz University of Medical Sciences, Karaj, Iran; 3 Department of Health Education & Promotion, Alborz University of Medical Sciences, Karaj, Iran; 4 Social Determinants of Health Research Center, Alborz University of Medical Sciences, Karaj, Iran; 5 Department of Midwifery, school of Medicine, Alborz University of Medical Sciences, Karaj, Iran

**Keywords:** Skin cancer, Sunscreen, Paddy workers, HAPA, Path analysis

## Abstract

**Background:**

Skin cancer is considered as one of the most common cancers in the world. There is little information about identifying factors affecting sunscreen use among paddy workers and their protective behavior. The present study aimed to determine a predictive model of the sunscreen use in the paddy workers based on the health action process approach model (HAPA).

**Methods:**

This cross-sectional study was conducted on 177 paddy workers who engaged in agricultural work in the north of Iran in 2018. Convenience sampling methods was used. Inclusion criteria were being a farmer for 5 years, working under the sunshine more than 2 h per day, and above the age of 30 years. A multi-sectional questionnaire (intention, risk perception (RP), outcome expectation (OE), action self-efficacy (ASE), action planning (AP), coping planning (CP), coping SE (CSE), self-monitoring (SM), and sunscreen use) was used for data collection. Data were analyzed with SPSS-21 and Lisrel-8.8 software.

**Results:**

The mean age of participants was 47.78 ± 12.66 years. The final path model fitted well (comparative fit index (CFI) = 0.98, RMSEA = 0.000), only coping self-efficacy (CSE) from both direct and indirect paths had an impact on sunscreen use (*B* = 0.73). Among the variables which are influenced only in one direction, coping planning (CP) had the most direct influence (*B* = 0.30) on behavior, and action planning had the lowest influence (*B* = 0.24).

**Conclusion:**

Coping self-efficacy was the most important factor which had influence on the use of sunscreen, and it should be considered when designing interventional programs related to sunscreen use among paddy workers.

## Introduction

Skin cancer is considered as one of the most common cancers in the world [1], and its prevalence is increasing. This cancer affects one of every five American people and leads to more than 10,000 deaths annually in the USA [2]. This cancer is also one of the most common types of cancers in Iran [3].

Ultraviolet (UV) radiation is considered as the most important cause of skin cancer [4]. Concerns related to occupational exposure to sunlight increase with the increase of skin cancer incidence [5, 6]. In relation to this cancer, the main emphasis is on outdoor jobs [7]. Farmers are among the most susceptible individuals to sunburn risk with consequent increase of the risk of skin cancer. While there is a little information about the factors affecting their performance and their protective behavior [8], there have been some studies conducted to understand the effective factors on the use of sunscreen, and a variety of factors have been suggested like risk perception [9], perceived sensitivity [10], self-efficacy [11], and outcome expectation [12].

The health action process approach model (HAPA) is considered as a predictive model for understanding the behavioral change mechanisms, and there are various experimental evidences to support this approach in different health behaviors such as healthy eating [13], vaccination [14], condom using [15], dental floss, physical activity, and management of diabetes [16].

The HAPA was first proposed by Schwarzer et al. [17] and is consisted of two phases named voluntary and motivational. The motivational phase focuses on beliefs that force a person to have particular behaviors and includes the factors such as Risk Perception (RP), Outcome Expectation (OE), and Action Self-Efficacy (ASE). These factors lead to the intention of behavior. After the formation of the intention, the person enters to the voluntary phase, which involves Action Planning (AP), Coping Planning (CP), and Coping Self-Efficacy (CSE) [18].

**Fig. 1:**
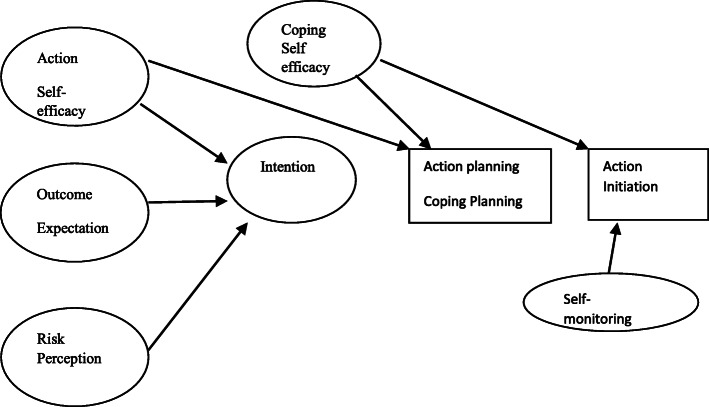
The default relationship between the variables, based on the health action process approach (HAPA)

Knowing the factors that affect the behavior, their importance and their direct and indirect effects of each of the variables will help planners and educators in designing appropriate educational interventions. In this regard, Craciun [18] conducted a study on female students by using the HAPA to identify the intermediary components of the use of sunscreen and showed that the planning variables just play a mediating role in the use of sunscreen for women. This study aimed to determine the predictive model of the sunscreen use for paddy workers by using HAPA (Fig. 1).

## Methods

### Study design and participants

This cross-sectional study was carried out on 177 paddy workers in 2018. These farmers were engaged in agricultural work in the villages of the Rood River in the north of Iran (Gilan province). Five of the 460 villages in Roudsar were selected by cluster random sampling to access the study subjects. The inclusion criteria were being a farmer for 5 years, working under the sunshine for more than 2 h per day, and above the age of 30 years. Farmers who were eligible for entrance in the study were selected by the convenience sampling method. By referring to the selected villages and agricultural lands, 354 farmers were surveyed in terms of inclusion criteria, and eventually 177 farmers entered the study.

### Sample size

For determining the sample size, usually, *N* = 100–150 is considered the minimum sample size for conducting path analysis [19].

For better access to the farmers, the researchers gets in contact with them in the agricultural land during the summer season of agriculture (from June till September). At the beginning, the study aims were explained to the subjects and farmers who were willing to participate in the study, and the written consent form was obtained. Each questionnaire was completed within 30 min approximately. Filling the questionnaire was conducted by interview, and the interviews were conducted by a trained interviewer (The first author: HP).

### Instruments

#### A multi-sectional questionnaire based on HAPA was used to collect data

The validity and reliability of the questionnaire was assessed by the content validity and the Cronbach’s alpha coefficient, respectively. For content validity, we used the opinions of 10 specialists (experts in the field). The Cronbach’s alpha will be presented when describing each component. This questionnaire included demographic characteristics, motivational factors (risk perception, outcome expectation, and action self-efficacy), and volitional factors (action planning, coping planning, coping self-efficacy, self-monitoring), intention, and sunscreen use as follows:

- Demographic characteristics and basic data related to sunscreen include age, sex, education, economic status, years of employment in farming, sunburn history, and a history of sunscreen.
- Intention: The individual decision to use sunscreen or not was assessed by two questions, e.g., “I plan to use a sunscreen with an appropriate SPF during the working under the sunlight”, furthermore, I intend to use sunscreen, during the working under the sunlight, also “I intend to renew it every two hours”. The Cronbach’s alpha coefficient calculated for this section was 0.89.
- Risk Perception (RP): RP was assessed by five questions, e.g., “When I am working under the sunlight without using sunscreen, there are possibilities of the occurrence of freckle and unpleasant appearance”. Higher scores represent more risk perception of ultraviolet (UV) and sunburn. The Cronbach’s alpha coefficient for this part was 0.82.
- Outcome Expectation (OE*)*: The benefits of using the sunscreen was evaluated by 4 statements. For example, “Using the sunscreen during the working under the sunlight makes my skin look fresher”, “Using the sunscreen during the working under the sunlight, will reduce sun burning and the itching”. The higher score represented more OE. The Cronbach's alpha coefficient for this part was 0.89.
- Action Self-Efficacy (ASE): The ability to use of sunscreen was assessed by three statements. For example, “I'm sure that I can use sunscreen during the working on agricultural land”. The higher score represented more ASE. The Cronbach's alpha coefficient for this part was 0.71.
- Action Planning (AP): AP was assessed by one statement; “I have planned to use sunscreen appropriately during the working under the sunlight, in specific times and locations”. The Cronbach’s alpha coefficient for this part was 0.71.
- Coping Planning (CP): CP was evaluated with three questions by considering the potential barriers, e.g., “I have plan to use sunscreen properly during the working under the sunlight at specific time, and specific location even if others ridicule me”, “I plan to use sunscreen properly while working under the sun at specific time and locations even if I face lack of time”. The Cronbach’s alpha coefficient for this part was 0.81.
- Coping Self-Efficacy (CSE): CSE was evaluated by a person’s belief about his own ability to overcome the obstacles in order to fulfill specific behavior. In this study, three main barriers for using sunscreen (distance, time limitation, and gender restrictions) were considered. These barriers were distinguished during a preliminary study, e.g., “I believe that despite the distance, I can buy sunscreen in the city when I am buying other supplies”, “I believe that I can use sunscreen in spite of gender restriction and ridicule by others”. The Cronbach’s alpha coefficient for this part was 0.81.
- Self-Monitoring (SM): The control of a person regarding the appropriate use of sunscreen was assessed by three statements, e.g., “I constantly monitor myself for using a sunscreen with a suitable SPF when I work in the sun”. The Cronbach’s alpha coefficient for this part was 0.70. The higher score indicates more control on the behavior.
- Sunscreen use: The behavior was examined by three statements, I regularly use sunscreen during the working on agricultural lands, “When I’m working on agricultural lands, I renew my use of sunscreen every two hours”, “When I am using sunscreen, I notice to its SPF (Sun Protection Factor )and its amount”.

The instrument questions were scored based on a 4-point Likert scale from strongly agree to strongly disagree*.* The questionnaire was filled by the participants in three occasions (at the beginning, a month later, and 2 months later).

### Ethical consideration

The present study was approved by the Ethics Committee of Alborz University of Medical Sciences (Ethical code: IR. ABZUMS. Rec. 1397.064), dated 05.08.2018.

### Data analysis

All data were analyzed by using SPSS software version 21 and LISRELS software version 8. First, the normality of the variables was evaluated using the Kolmogorov–Smirnov test.

The significance of correlation between variables was considered as the first hypothesis of path analysis. The intention, RP, OE, ASE, AP, CP, CSE, and SM were considered as independent variables, and sunscreen use was considered as a dependent variable. In order to evaluate the fitness of the model, the fitting index such as x2/df, root mean square error of approximation (RMSEA), comparative fit index (CFI), goodness of fit index (GFI), and normal fit index (NFI) were computed.

## Results

### Characteristics of participants

The mean age of the participants was 47.78 ± 12.66 years, which ranged from 30 to 79 years. Average years of employment in agriculture were 18.67 ± 11.63. The majority of the subjects were women (69.3%), at the age of 40–30 years (40.68%), and 45.2% of them have diploma. Most of the people who participated in this study had 5–10 years of work experience (38.42%). Positive sunburn history was reported by 93.9%, and history of the sunscreen use was 60.45%. The socioeconomic status of the majority (79.3%) was inadequate (Table 1).

**Table 1:** The demographic characteristics of the (*n* = 177) Iranian’s paddy workers in 2018

Variable	***N*** (%)
**Age (mean ± SD)**	47.12 ± 78.66
30–40	69 (38.99)
41–50	43 (24.29)
51–60	34 (19.21)
> 60	31 (17.51)
**Education**	
> 12	64 (36.16)
12	80 (45.20)
< 12	33 (18.64)
**Gender**	
Male	53 (29.94)
Female	124 (70.06)
**Sunburn**	
Yes	168 (94.92)
No	9 (5.08)
**Farming history (mean ± SD)**	18.67 ± 11.63
5–10	68 (38.42)
11–20	60 (33.90)
21–30	26 (14.69)
> 30	23 (12.99)
**Sunscreen use**	
Yes	107 (60.45)
No	70 (39.55)
**Economic status**	
High	3 (1.70)
Moderate	32 (18.08)
Low	142 (80.22)

The mean and the standard deviation of RP, OE, and ASE in this study respectively were 14.06 ± 3.65, 8.09 ± 2.34, and 7.12 ± 2.36. Table 2 displays the mean and the standard deviation of the other constructs.

**Table 2:** Mean and standard deviation of the construct of HAPA (*n* = 177)

Variable	Mean	SD	Min	Max
RP	14.06	3.65	5	20
OE	8.09	2.34	3	12
ASE	7.12	2.36	3	12
AP	2.64	0.83	1	4
CP	6.84	2.34	3	12
CSE	6.84	2.37	3	12
SM	9.06	3.03	4	16
Sunscreen use	4.28	1.64	3	12

*RP* Risk Perception, *OE* Outcome Expectation, *ASE* Action Self-Efficacy, *AP* Action Planning, *CP* Coping Planning, *CSE* Coping Self-Efficacy, *SM* Self-monitoring

The correlation between the study variables is shown in Table 3. The strongest correlation was between CSE and SM.

**Table 3:** Correlations between RP, OE, ASE, CSE, AP, CP, and SM; intention; and behavior

	RP	OE	ASE	AP	CP	CSE	SM	Behav	Intent
RP	1								
OE	0.81**	1							
ASE	0.630**	0.785**	1						
AP	0.647**	0.726**	0.667**						
CP	− 0.536**	0.670**	0.721**	0.688**	1				
CSE	0.545**	0.691**	0.748**	0.701**	0.902**	1			
SM	0.510**	0.625**	0.729**	0.638**	0.849**	0.851**	1		
Sunscreen	0.065	0.208**	0.122*	0.168	0.144	0.210**	0.130	1	
Intent	0.610**	0.726**	0.792**	0.677**	0.825**	0.820**	0.813**	0.157*	1

*Significant at level 0.05; **Significant at level 0,01

*RP* Risk Perception, *OE* Outcome Expectation, *ASE* Action Self-Efficacy, *AP* Action Planning, *CP* Coping Planning, *CSE* Coping Self-Efficacy, *SM* Self Monitoring, *Intent* Intention

### Structure model

**Fig. 2:**
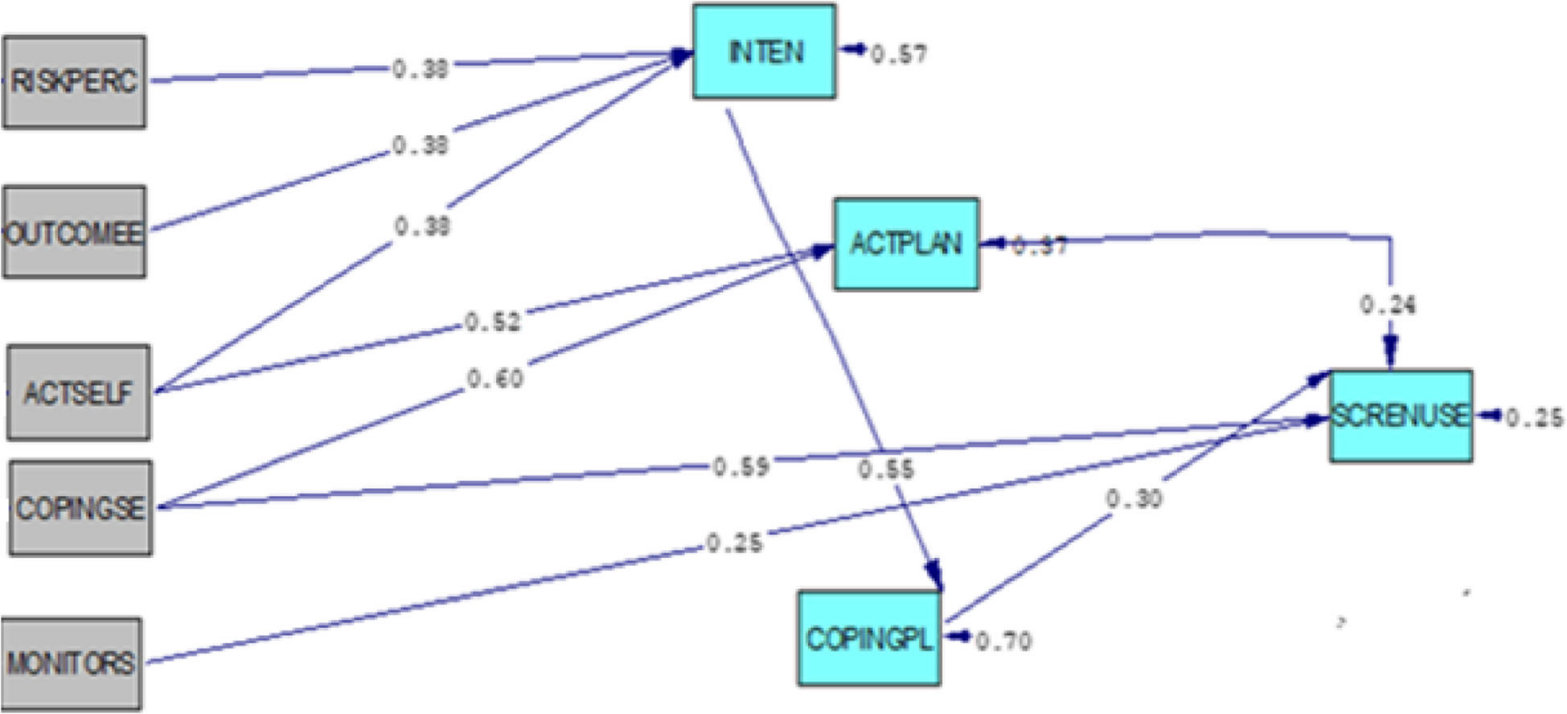
Final path analysis model. RISKPERC: Risk Perception; OUTCOMEE: Outcome Expectation; ACTSELF: Action Self Efficacy; COPINGSE; Coping Self-Efficacy; MONITORS: Self – Monitoring; INTEN: Intention; ACTPLAN: Action Planning; CSE: Coping Planning; SCRENUSE: Sun Screen Use

Based on the final model (Fig. 2), among the variables which are influenced only in one direction, CP had the most direct association with behavior and AP had the lowest association. In an indirect route, ASE and the intention had the most relationship with behavior; RP, and OE together had equal and the lowest association. Only CSE had direct and indirect paths. All the pathways are shown in Table 4.

**Table 4:** Direct and indirect associations between HAPA variables and sunscreen use

Variable	Direct effect	Indirect effect	Total effect	*t* value (for direct)	*R*^2^
RP	-	0.0627	0.0627	4.33	0.65
OE	-	0.0627	0.0627	4.33	
Action SE	-	0.1875	0.1875	6.24	
Coping SE	0.59	0.144	0.734	18.35	
SM	0.25	-	0.25	6.54	
Intent	-	0.165	0.165	-	
AP	0.24	-	0.24	-	
CP	0.30	-	0.30		

*RP* risk perception, *OE* outcome expectation, *Action SE* action self-efficacy, *Coping SE* coping self-efficacy, *CSE* coping self-efficacy, *SM* self-monitoring, *Intent* intention, *AP* action planning, *CP* coping planning

## Discussion

Due to the final fitted model, AP and CP were two variables, which are directly affecting the behavior of paddy workers.

Consistent with the current study finding, de Vries et al.’s study showed that AP was the strongest predictor of sunscreen use in Belgian teens [20]. Planning plays an important role in the process of changing behavior and communicates between the intention and the behavior. AP is more applicable in the early stages of behavior change, and coping planning is more applicable in the next stages of behavior change [21]. In this study, both the AP and CP have been assessed together, and separately assessing these variables was impossible. Although, some studies concluded that together, those two variables are essential in changing behavior [22], and it is believed that CP can boost the effects of AP [23].

The coping SE was the only variable that had both direct and indirect paths on the behavior and had the greatest effect on sunscreen use among farmers, which is the total of the direct and indirect, based on the final fitted model. CSE is mentioned as a personal SE to overcome the barriers. During the several situations, maintaining health behavior was harder than starting it, although for starting health behavior, ASE is sufficient, but for maintaining it, CSE is required [24].

In a Nahar et al. study, ASE had a significant relationship with protective behaviors against the sunlight in landscapers [25]. Although, in the study of Nahar, it has not mentioned anything about the continuation and preservation of protective behaviors, and therefore, we cannot compare these two elements.

Based on the final fitted model, there was no direct relationship between intention and behavior; intention goes indirectly through the CP path on behavior, which is the same as the Craciun study that was planning a variable between the intention and sunscreen use among students. Based on the Craciun study, having a good intention leads to behavior, when we have the appropriate planning to overcome the barriers [18].

According to Rhodes & de Bruijn’s study, intention determines 46% variation in behavior [26]; but despite having good intentions,^,^ many planners are failing to conduct the behavior [27]. And the intention has the limited predictive power [28], contrary to the planned behavior model and protection motivation theory assumptions, which considered intention as the strongest predictor of behavior. According to Rhodes & Dickau, declaration of the intention was an essential factor for behavior, but it is not enough [29]. Planning will increase the possibility of converting the intention to behavior [30].

According to Osch et al.’s study, their results showed that the motivational factors such as RP, OE, and ASE did not directly affect behavior [31].

In accordance with these study results, the effects of RP and OE were the same as in predicting sunscreen use and was less than ASE. While the Craciun [18] study represented that RP was less important in comparison with OE and ASE in the sunscreen use among students; it seems that the different results are due to the differences in the subject’s characteristics; younger people often have less cautious behaviors and less risk perception compared with older people, and the outcome expectation is more important to them than older ones. Both in this study and in Craciun’s study [18], SE was more important than the other motivational factors. In current studies, like Craciun’s study, ASE was more important than RP or OE. As this study was carried out for people who are over 30 years with a mean age of 47 years old, there is no definite opinion on this subject for researchers, until this study was conducted

In this study, SM has direct influence on the behavior. This variable in fact is a facilitator of changing the behavior, as Sniehotta believes that in addition to AC and CP, we need strategies such as social support and SM for changing behavior [32].

### Limitations of the study

Given that the current study was conducted in the agricultural season, some factors such as farmers lacking time for interview might influence data collection cycle although we attempted to adjust the interview time in accordance with the participant’s conditions.

## Conclusions

Coping SE is the most important factor which had influence on sunscreen use, and it should be considered in designing interventional study related to sunscreen use among paddy workers. Furthermore, it should be noticed that the motivational factors are not sufficient, but we should focus on the planning factors alongside the motivational factors in changing behavior, in order to promote sunscreen use in farmers.

## Data Availability

All datasets in this study are available on reasonable request.

## Abbreviations

HAPA: Health action process approach
RP: Risk perception
OE: Outcome expectation
ASE: Action self-efficacy
AP: Action planning
CP: Coping planning
CSE: Coping self-efficacy
SM: Self-monitoring
RMSEA: Root mean square error of approximation
CFI: Comparative fit index
GFI: Goodness of fit index
NFI: Normal fit index
IFI: Incremental fit indices
